# Tumour suppressor PTEN activity is differentially inducible by myo‐inositol phosphates

**DOI:** 10.1111/jcmm.17699

**Published:** 2023-02-27

**Authors:** Krzysztof Grzymajlo, Bouchra El Hafny‐Rahbi, Claudine Kieda

**Affiliations:** ^1^ Department of Biochemistry and Molecular Biology, Faculty of Veterinary Medicine Wroclaw University of Environmental and Life Sciences Wroclaw Poland; ^2^ Centre for Molecular Biophysics UPR 4301 CNRS Orleans France; ^3^ Military Institute of Medicine Laboratory of Molecular Oncology and Innovative Therapies Warsaw Poland

**Keywords:** angiogenesis, cancer, hypoxia, ITPP, PTEN

## Abstract

Tumour evolution and efficacy of treatments are controlled by the microenvironment, the composition of which is primarily dependent on the angiogenic reaction to hypoxic stress. Tumour angiogenesis normalization is a challenge for adjuvant therapy strategies to chemo‐, radio‐ and immunotherapeutics. Myo‐inositol trispyrophosphate (ITPP) appears to provide the means to alleviate hypoxia in the tumour site by a double molecular mechanism. First, it modifies the properties of red blood cells (RBC) to release oxygen (O_2_) in the hypoxic sites more easily, leading to a rapid and stable increase in the partial pressure of oxygen (pO_2_). And second, it activates the endothelial phosphatase and tensin homologue deleted on Chromosome 10 (PTEN). The hypothesis that stable normalization of the vascular system is due to the PTEN, a tumour suppressor and phosphatase which controls the proper angiogenic reaction was ascertained. Here, by direct biochemical measurements of PTEN competitive activity in relation to PIP2 production, we show that the kinetics are complex in terms of the activation/inhibition effects of ITPP with an inverted consequence towards the kinase PI3K. The use of the surface plasmon resonance (SPR) technique allowed us to demonstrate that PTEN binds inositol derivatives differently but weakly. This method permitted us to reveal that PTEN is highly sensitive to the local concentration conditions, especially that ITPP increases the PTEN activity towards PIP3, and importantly, that PTEN affinity for ITPP is considerably increased by the presence of PIP3, as occurs in vivo. Our approach demonstrates the validity of using ITPP to activate PTEN for stable vessel normalization strategies.

## 1 INTRODUCTION

Tumour treatment issues are due to the tumour microenvironment Thus, it must be considered in order to produce efficient therapies.^1^, ^2^, ^3^ The causative parameter from which the characteristics of the microenvironment result is the establishment of hypoxia in a growing tumour and the adaptation of the cells to hypoxic stress.^4^, ^5^ In terms of secreted factors and phenotype, cellular properties are modulated by an early response to hypoxic stress.^6^

Apart from the tumour cells that first have to adapt to the lack of oxygen, the recruited immune and stromal cells originally activated to fight tumour development through an active immune response get conditioned to such stress and participate in hypoxic tumour expansion and aggressiveness.^7^, ^8^, ^9^

Angiogenesis is the first response to hypoxia by the HIF‐1 and ‐2 transcription in the tumour cells, leading to the synthesis of proangiogenic factors responsible for typical pathologic tumour angiogenesis, which is the first hallmark of cancer and from which other hallmarks result.^8^, ^9^

Although aiming to compensate for the low partial pressure of oxygen inside the tumour, hypoxic stress‐induced angiogenesis is inefficient due to the overproduction by the tumour cells of vascular endothelial growth factor (VEGF) along with other factors.^10^, ^11^, ^12^, ^13^ The newly formed pathological vessels not only are unable to sustain the blood flow but also favour the dissemination of hypoxia‐ and acid‐resistant tumour cells.^10^ Moreover, it has been widely documented that hypoxia‐conditioned factors signal the recruitment of immunosuppressive cells or modulate the phenotype of immune cells in the microenvironment, making them more tolerant to tumour cells.^14^, ^15^ Consequently, the challenge is to make the tumour vessels functional to instal a blood flow that allows for the delivery of treatment molecules^11^, ^16^ and allows the red blood cells to increase the pO_2_, thus, alleviating hypoxia.^17^, ^18^ It is therefore necessary to disrupt the equilibrium of haemoglobin‐mediated O_2_ release, allowing the VEGF pathway to be downregulated as it is responsible for turning on and maintaining the proangiogenic state. The pO_2_ increase in the vessels, due to the dissociation of the red blood cell carried‐oxyhaemoglobin, provides the signals for primary hypoxia alleviation/vessel normalization.^19^, ^20^ As shown, such an effect can be reached transiently by antiangiogenic strategies aiming to eliminate the proangiogenic factors overexpressed in the tumours. The limitations were mainly due to the poorly applicable controls of such active molecules that lead to the destruction of the vessels and the deleterious selection of aggressive tumour stem‐like cell populations.^10^, ^21^, ^22^ The direct modification of the properties of haemoglobin (Hb) by an allosteric effector makes it possible to shift its dissociation curve, allowing for the easier and complete release of O_2_ in equilibrium with the pO_2_ external to the erythrocytes.^23^, ^24^, ^25^ Myo‐inositol tris pyrophosphate (ITPP) was shown to operate this pO_2_ increase upon the direct modification of the RBCs.^24^, ^26^ Its use in the angiogenesis‐based treatment of hypoxia‐dependent diseases such as cancer^19^ or heart failure^27^, ^28^ has shown that its long‐term administration led to stable vessel normalization. This has resulted in the hypothesis being propounded according to which its action is dependent on phosphate‐linked pathways.^19^ The most significant is the action of the phosphatase and tensin homologue mutated on chromosome 10 (PTEN), mutated in multiple advanced cancers.^29^, ^30^ PTEN is the main tumour suppressor the activation of which controls the PI3K/AKT/mTOR pathway in the cytoplasm and the nucleic p53 suppressive activity.^31^ Moreover, the significance and potential of PTEN as a key suppressor of tumour growth is underlined by its major role in orchestrating angiogenesis progression. More precisely, PTEN controls the NOTCH4‐mediated regulation of the tip‐stalk organization of endothelial cells to build normal vessels.^32^ In view of the necessarily active PTEN in normal vessels, it makes it a highly potent candidate for a vessel normalization target.^20^, ^32^, ^33^

Besides the effect of ITPP‐charged RBCs on angiogenesis in vitro, resulting directly from the release of oxygen under shear stress,^26^ the ITPP molecule does activate the endothelial PTEN.^19^ In vivo, upon ITPP injection, both a rapid pO_2_ increase inside the tumour, as well as a long‐term bioavailability of ITPP in the blood, were observed in the treatment protocols. This indicates that such an approach may lead to stable vessel normalization, offering an adjuvant strategy for the treatment of the numerous hypoxia‐dependent diseases.^19^

Tumour reaction analysis demonstrated that ITPP‐mediated vessel normalization in tumours can modify the microenvironment so strongly that the cells expressing PD‐L1 and its level of expression were downregulated. Likewise, CTLA4 and CD47 were reduced, while PD1‐expressing cells increased.^20^ The Tregs were less present and the M1 macrophage phenotype increased. NK cell entry into the tumour mass was shown to be regulated by the level of PD‐L1 expression on the tumour vessel endothelial cells, which is induced in hypoxia but down‐regulated upon hypoxia alleviation and PTEN activation.^20^

As immunotherapy is the actual main purpose for cancer treatment, strategies that favour the immune response are actively searched for, with a huge need for methods that would be independent of the inhibitory monoclonal antibodies specific for either PD1 or PD‐L1 that unfortunately do not treat the causal mechanisms of their induction; thus, this limits the impact of their use.^34^

Our approach allows for the modification of the tumour microenvironment by taking advantage of the Hb allosteric properties and their enhancement by ITPP for a complete O_2_ release in order to, on the one hand, alleviate hypoxia in the pathological sites and, on the other hand, for its long‐term action on endothelial cell PTEN activation to maintain the vessels in their normal state, ensuring a prolonged blood flow efficacy. Consequently, the oxygen level inside the tumour is continuously maintained in physioxia. This breaks the vicious circle in which pathologic angiogenesis is responsible for keeping a tumour hypoxic, demonstrating that PTEN activity in the endothelial cells is critical.^5^, ^35^

Remarkably, such an approach presents the main advantages of PTEN expression and activity being independent of the properties of tumour cell. This strategy is addressed to the endothelial cells that perform angiogenesis, which are external normal cells recruited by hypoxia‐responding tumour cells. Indeed, vessel normalization by ITPP only deals with the normal endothelial PTEN to activate it. Consequently, ITPP treatment allows for tumour therapy regardless of the possible mutations of the PTEN tumour suppressor gene. Moreover, considering the numerous ways in which PTEN can exert its tumour suppressor effect, ranging from the direct long form of soluble PTEN interacting with PTEN^**−**^ cells to the exosome‐specific microRNA (miRNA) modulation, the proposed strategy of its activation possesses a huge potential for improving the efficacy of anti‐tumour approaches.^36^

In view of this, the present work was undertaken to decipher whether the direct recognition of ITPP by PTEN occurs, if such an interaction can be specific compared to ITPP with its metabolite, the bisphosphate bis‐pyrophosphate (BPBPP) and the inositol hexaphosphate (IHP) with the non‐cyclic form of phosphate residues. The biochemical assessment of the modulation of PTEN activity by those molecules was compared to the similar modulation of PI3K, the kinase that produces PIP3 to activate the proliferation pathway, which showed an interdependency of both the phosphatase and the kinase with regards to the action of ITPP in a highly concentration‐dependent manner.

Moreover, the biological reaction of endothelial cells to the process of hypoxia/reoxygenation evidenced that the activation of PTEN is greatly assisted by ITPP and involves the AKT activation pathway.

Direct evidence of PTEN/ITPP recognition and binding was found here using the plasmon resonance technique to assess the binding characteristics of possible PTEN substrates. It allowed the direct but weak binding of ITPP and BPBPP to be displayed by the enzyme, as compared to the natural substrate PIP3, and a high increase of this binding when PIP3 and ITPP interact with PTEN. Based on the present data, ITPP and BPBPP enhance PIP3 binding by PTEN specifically, and such an activation effect is highly concentration‐dependent.

When experiments were performed in conditions mimicking the intracellular environment where PTEN is in the presence of PIP3, we observed a much more efficient association for ITPP which was able to bind strongly to a PTEN‐PIP3 preformed complex, similar to the in situ situation. The data indicate that ITPP provides a potential mean to induce PTEN activation and control cell growth in hypoxic pathologic environments.

## 2 MATERIALS AND METHODS

### 2.1 Effect of ITPP treatment on the activation of endothelial PTEN

Frozen sections of murine melanoma tumours treated as previously described^19^ were labelled by anti‐PTEN (rabbit IgG) (Cell Signalling) and mouse anti‐CD31 (rat monoclonal IgG2a) (eBiosciences) before detection by tetramethylrhodamine isothiocyanate and fluorescein isothiocyanate secondary antibodies, respectively. Nuclei were stained with bis‐Benzimide H 33258 (Sigma‐Aldrich).

Fluorescent microscopy visualization was performed on a Zeiss 200 M inverted fluorescence microscope (Le Pecq, France), a video microscopy station with a controlled temperature, hygrometry and gas composition. Analyses were carried out using the Axiovision software.

### 2.2 PTEN phosphatase activity‐mediated phosphate production assessment towards ITPP, BPBPP and IHP

The malachite green‐molybdate reagent was used for a colorimetric assay of pure PTEN phosphatase activity (MAK308, Sigma‐Aldrich). The colorimetric assay was used to examine PTEN interactions with ITPP and BPBPP (about 90% enriched fraction, synthetized by Prof. Jean‐Marie Lehn and coworkers), both kindly provided by Professor J‐M. Lehn, Institut de Science et d'Ingénierie Supramoleculaires (ISIS), University of Strasbourg, 67,000 Strasbourg, France, and IHP (from Sigma‐Aldrich, Ca14306‐25‐3) regarding its activity on PIP3 as inhibitors or activators (40). The formed complex between malachite green molybdate and free orthophosphate absorbs at 620–640 nm. The PTEN substrate: water‐soluble phosphoinositide (Cell Signals, Lexington, KY, USA) was used for positive control and standardization. For a 96‐well plate assay format, 1.0 to 4 × 10^−6^ g of recombinant PTEN protein (SRP4838‐5UG from Sigma‐Aldrich) 0.25 to 1.0 × 10^−6^ M were incubated with the PIP3 substrate (from 50 × 10^−6^ M to 200 × 10^−6^ M) in 50 μL of Tris–HCl (pH 7.5) buffer with 10 mM dithiothreitol (DTT) for 1 h at 37°C; the reaction was terminated by the addition of 100 μL of malachite green reagent (Biomol Green, AK‐111) for 15 min (25°C), and the formed complex was measured at 620 nm. The amount (nmol) of free phosphate released in each well was determined by linear regression analysis against a standard phosphate curve.

### 2.3 PTEN activity towards ITPP, BPBPP and IHP assessed by PIP2 production

PTEN biochemical activity towards ITPP, BPBPP and IHP was assessed by an ELISA competition assay using the lipid Phosphatase Activity Assay to quantify PTEN activity (ELISA kit K‐4700, Echelon Biosciences, Salt Lake City, UT) following the manufacturer's protocol, designed to quantify the phosphatase activity of PTEN by the detection of the enzyme product, PI(4,5)P_2_. Reagents are added to a PI(4,5)P_2_‐coated microplate, the concentration of which is detected by the PI(4,5)P_2_ detector protein added for competitive binding and, in turn, detected by a peroxidase‐linked secondary detector. The quantitative colorimetric signal is inversely proportional to the amount of PI(4,5)P_2_ produced by PTEN. Recombinant PTEN (E‐3000; Echelon Bioscience) 1 ng/μL (13 × 10^−9^ M) was allowed to react with PI(3,4,5)P_3_ (5 × 10^−6^ M) to produce PI(4,5)P_2_, in the presence or not of ITPP, BPBPP or IHP (concentration range 0.1 × 10^−6^ M to 100 × 10^−6^ M). PTEN was incubated with myo‐inositol derivatives (concentration range from 0.1 × 10^−6^ M to 100 × 10^−6^ M) for 15 min at 37°C, then, the PI(3,4,5)P_3_ substrate was added for a final concentration of 5 × 10^−6^ M, and the reaction was incubated at 37°C for 2.25 h before the detection steps.

### 2.4 PI3‐Kinase activity towards ITPP assessed by PIP3 production

PI3‐Kinase biochemical activity towards ITPP, BPBPP and IHP was assessed by an ELISA competition assay. Class I phosphoinositide 3‐kinase (PI3‐K) activity is measured through their reaction product, PtdIns(3,4,5)P_3_ (PIP3) using the PI3‐Kinase activity ELISA kit (Echelon. K‐1000 S). PI3‐K reactions are run with PI(4,5)P_2_ (PIP2). The enzyme, the PIP3 standards, and the controls were mixed and incubated with highly specific PIP3 binding and then transferred to a PIP3‐coated microplate for competitive binding with the PIP3 detector protein, further detected by a peroxidase‐linked secondary detector and quantitatively evidenced by a colorimetric reaction to estimate the amount of PIP3 produced. The quantitative colorimetric signal at 560 nm was inversely proportional to the amount of PI(3,4,5)P_3_ produced by PI3K. Recombinant PI3K was allowed to react with PI(4,5)P_2_ (5 × 10^−6^ M) to produce PI(3,4,5)P_3_ in the presence or not of ITPP, BPBPP or IHP (concentration range 0.1 × 10^−6^ M to 100 × 10^−6^ M).

### 2.5 Endothelial cell PTEN modulation and phospho‐AKT activation upon hypoxia/reoxygenation stress; the effect of ITPP and BPBPP

#### 2.5.1 Endothelial cells

FVB mouse lung microvascular endothelial cells (MLuMEC, FVB)^37^, ^38^, ^39^ were routinely cultured in Opti‐MEM/2% FBS, in a humidified incubator in 19.5% oxygen and were oxygen deprived in a Biotronix incubator allowing pO_2_ regulation (1%) and a time setting.

The schematic representation of the culture in hypoxic vs normoxic conditions to assess the effect of ITPP and BPBPP on the PTEN/PI3K/AKT pathway is provided below:

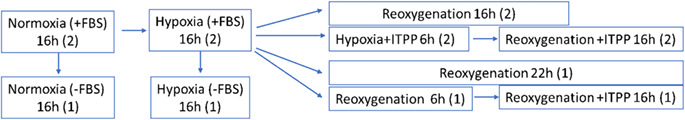

For flow cytometric detection, the cells were permeabilized by Fix and Perm Cell Fixation and the Permeabilization Kit ab185917 (Abcam), and were further labelled by rabbit anti‐PTEN mAb IgG, or rabbit anti‐phospho‐AKT mAb IgG (Cell Signalling Technology), and revealed by FTC‐labelled anti‐Rabbit IgG antibodies (R&D Systems). Flow cytometry experiments were conducted using the FACSCalibur cytometer and the CellQuest software for analysis (Becton–Dickinson, USA).

### 2.6 Real‐time interaction analysis by surface plasmon resonance

The binding of different analytes (PIP3, ITPP, BPBPP, IHP) and their mixtures to recombinant PTEN immobilized on CM5 sensor chips (GE Healthcare) was analysed by surface plasmon resonance (SPR) using a BIAcore T200 system. The PTEN immobilization level corresponds to approximately 5500 RU. All the binding experiments were carried out at 25°C with a flow rate of 30 μL/min in HBS running buffer.

To determine the affinity of the analysed ligands and their mixtures to PTEN, at least five different concentrations of each as well as a sample buffer blank were passed over the ligand‐immobilized chip surface (association phase), followed by dissociation with running buffer (HBS). Details of each set of interactions are provided in the table below (Table 1). The same samples were passed over a control chip surface without an immobilized ligand. Three replicates of each analyte concentration were injected. The resulting sensorgrams were obtained by first subtracting the buffer blank from the curves recorded for the interactions of PIP3 with PTEN. Then, the curves recorded when PIP3 was passed over the blank surface were subtracted from such sensorgram. The equilibrium constants were determined using BIAevaluation 3.1 software. For global fitting, a 1:1 Langmuir binding model with an included mass transport step was applied based on the criteria provided by the BIAevaluation handbook.

**TABLE 1:** Surface plasmon resonance analysis conditions.

SPR Experiment	Immobilized Ligand	Analyte	Analyte concentration	Association time (s)	Dissociation time (s)
1	PTEN	PIP3	25–400 μM	120	600
2	PTEN	ITPP or BPBPP or IHP	20–100 mM	120	600
3	PTEN	PIP3 and ITPP or BPBPP or IHP mixture	12.5–200 μM and 20 mM	120	600
4	PTEN	ITPP/BPBPP followed by PIP3	5–100 mM followed by 12.5–200 μM	360 followed by 480	600
5	PTEN	PIP3 followed by ITPP or BPBPP	200 μM, followed by 5–100 mM	480 followed by 480	1500

### 2.7 Statistical analysis

The data are presented as the mean ± SEM of at least three independent experiments The analysis was performed using GraphPad Prism software. Student's *t*‐test or anova was used to assess the statistical significance. The data were presented as means ± SEM. Differences were considered statistically significant at *p < 0.05 or **p < 0.01.

## 3 RESULTS

### 3.1 Activation of PTEN upon in vivo treatment by ITPP

**FIGURE 1:**
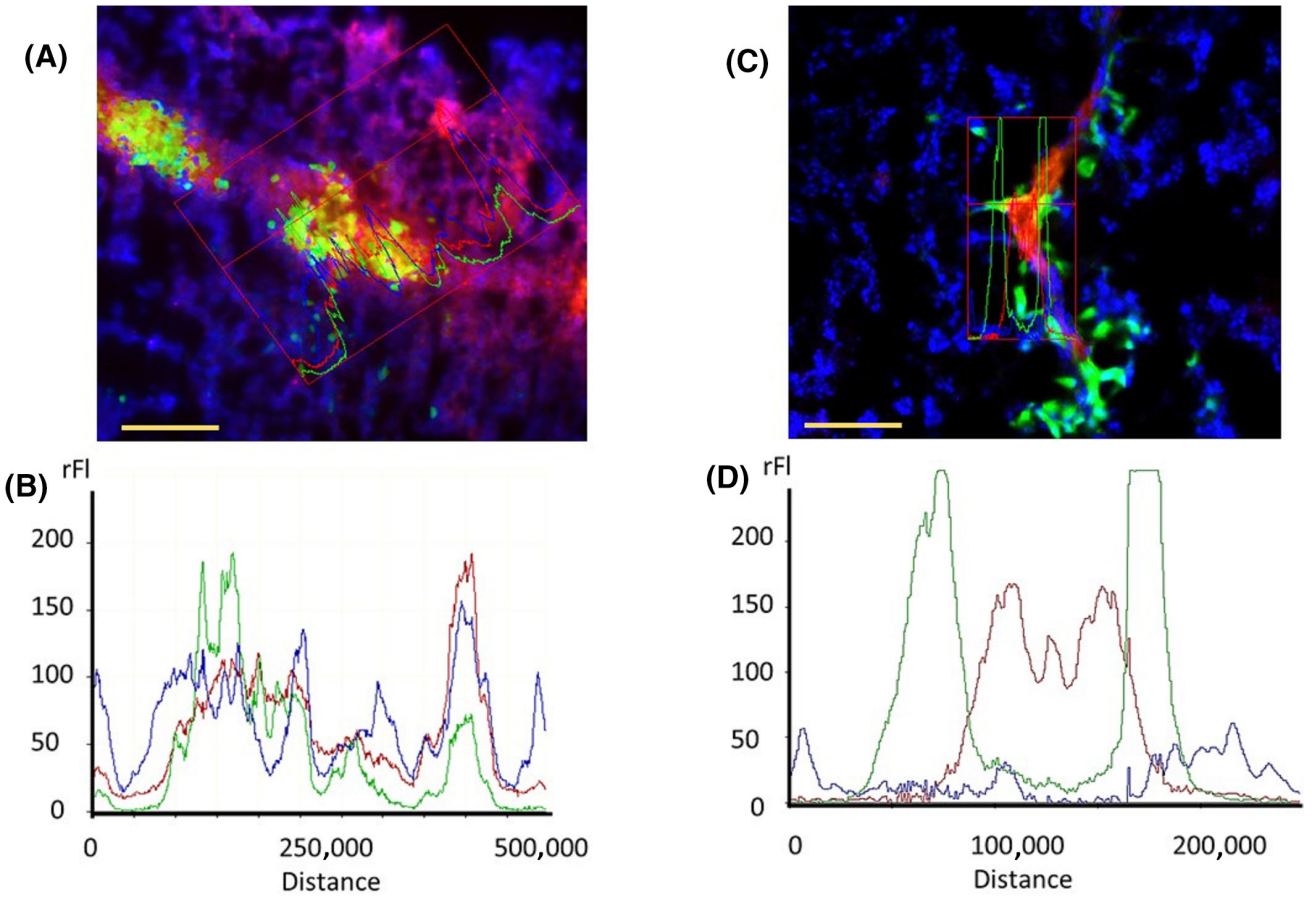
Localization of endothelial PTEN vs CD31 in tumour vasculature‐associated cells before (A, B) and after ITPP treatment (C, D). Fixed frozen sections of B16F10 melanoma tumours were treated for PTEN detection by anti‐PTEN rabbit IgG and anti‐CD31 rat IgG2a, detected by TRTC‐ and FTC‐labelled secondary antibodies, respectively. The nuclei were stained with bisbenzimide H33258. The a to d panels display the PTEN and CD31 labelling before (A, B) and after (C, D) ITPP treatment. Image analysis was performed using the AxioVision software to evidence colocalization (B) in non‐treated tumours (A) and the redistribution of PTEN in CD31 cells after vessel normalization (C, D). Scale bars = 100 μm.

Microscopic analyses of the vasculature status in B16F10 melanoma tumour‐bearing animals,^19^ as presented in Figure 1A, show the CD31‐positive endothelial cells (ECs) randomly distributed among the tumour tissue before the treatment of the melanoma‐bearing animals with ITPP. Such EC distribution, typical for pathologic angiogenesis and characteristic of the hypoxic growing solid tumours, indicates the colocalization of CD31 with the PTEN protein, as analysed in Figure 1B. After the ITPP treatment, according to the previously described protocol,^19^, ^28^ PTEN protein distribution was distinct from CD31 (Figure 1C, D), which clearly indicated a PTEN activation state.^40^, ^41^ This effect correlates with the restructuring of the vessel morphology, which appears normalized upon ITPP treatment (Figure 1C), as opposed to chaotic in the untreated tumours (Figure 1A).

### 3.2 Effect of myo‐inositol phosphate derivatives on PTEN hydrolytic activity towards PIP3

#### 3.2.1 PTEN phosphatase activity towards ITPP, BPBPP and IHP

**FIGURE 2:**
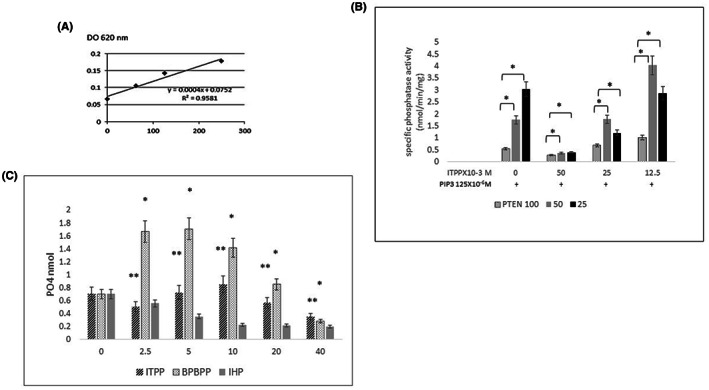
PTEN phosphatase activity by measurement of phosphate production for the assessment of the interaction between myo‐inositol derivative and PTEN. (A) Dose–response curve for phosphate concentration measured by absorbance at 620 nm of molybdate green/orthophosphate complex formation. (B) Soluble PIP3 (125 × 10^−6^ M) hydrolysis by PTEN (25 × 10^−6^ to 100 × 10^−6^ M) performed in the presence of variable concentrations (12.5 × 10^−3^ to 50.0 × 10^−3^ M) of ITPP. (C) Myo‐inositol derivative relative reactivity with PTEN. Soluble PIP3 (200 × 10^−6^ M) hydrolysis by PTEN (50 × 10^−6^ M) in the presence of variable concentrations (2.5 × 10^−3^ to 40.0 × 10^−3^ M) of ITPP, BPBPP or IHP. The data are the means of three separate experiments in triplicate. The data are the means ± SD of three separate experiments in triplicate, *p < 0.05, **p < 0.01.

Malachite green molybdate/orthophosphate complex formation measured after the PTEN hydrolytic reaction on PIP3 was performed in the presence or absence of myo‐inositol phosphate derivatives as possible modulators of the enzyme.^42^ The formed complex was measured at 620 nm. The amount (nmol) of free phosphate released in each well was determined by linear regression analysis against a standard phosphate curve (Figure 2A).

Figure 2A displays the dose–response curve assessing for phosphate concentration, as measured by the absorbance at 620 nm of the molybdate green/orthophosphate complex formation. Figure 2B shows the soluble PIP3 (125 × 10^−6^ M) hydrolysis by PTEN (25 × 10^−6^ M to 100 × 10^−6^ M) performed in the presence of variable concentrations (12.5 × 10^−3^ to 50.0 × 10^−3^ M) of ITPP. It indicates that the ratio of PIP3 to PTEN concentrations dictates the relative activity of PTEN towards its natural substrate. 125 × 10^−6^ M PIP3 is more actively hydrolysed by 25 × 10^−6^ M PTEN than by higher enzyme concentrations. This is confirmed by the activity measured in the presence of ITPP. Indeed, a lower ITPP concentration is associated with phosphate production by 50 × 10^−6^ M PTEN while it has no action on the hydrolytic activity exerted by 25 × 10^−6^ M PTEN towards PIP3 (125 × 10^−6^ M). Similarly, no effect is observed for a higher PTEN concentration (100 × 10^−6^ M). Higher concentrations of ITPP (25 × 10^−6^ M and 50 × 10^−6^ M) appeared to be inhibitory for all the tested PTEN concentrations. Figure 2C presents the phosphate‐releasing activity of PTEN by hydrolysis of PIP3 and the effect of the presence of inositol phosphate derivatives. PIP3 (200 × 10^−6^ M) was hydrolysed by PTEN (50 μM) in the presence or not of various concentrations (2.5 to 40 × 10^−3^ M) of ITPP, BPBPP or IHP. While IHP inhibits hydrolysis in a concentration‐dependent manner, BPBPP increases phosphate production either by the direct hydrolysis of the BPBPP itself or through the enzyme activation by BPBPP for concentrations ranging from to 2.5 to 20 × 10^−3^ M, as a higher BPBPP concentration (40 × 10^−3^ M) inhibits phosphatase activity. Used at similar concentrations, ITPP alone displays moderate hydrolytic susceptibility, which is reduced when ITPP concentration reaches 40 × 10^−3^ M. When combined with PIP3, ITPP inhibits phosphatase activity but indicates that there is a complex induction/inhibition mechanism.

#### 3.2.2 Effect of myo‐inositol phosphate derivatives on PTEN phosphatase activity towards PIP3 and PIP2 production

**FIGURE 3:**
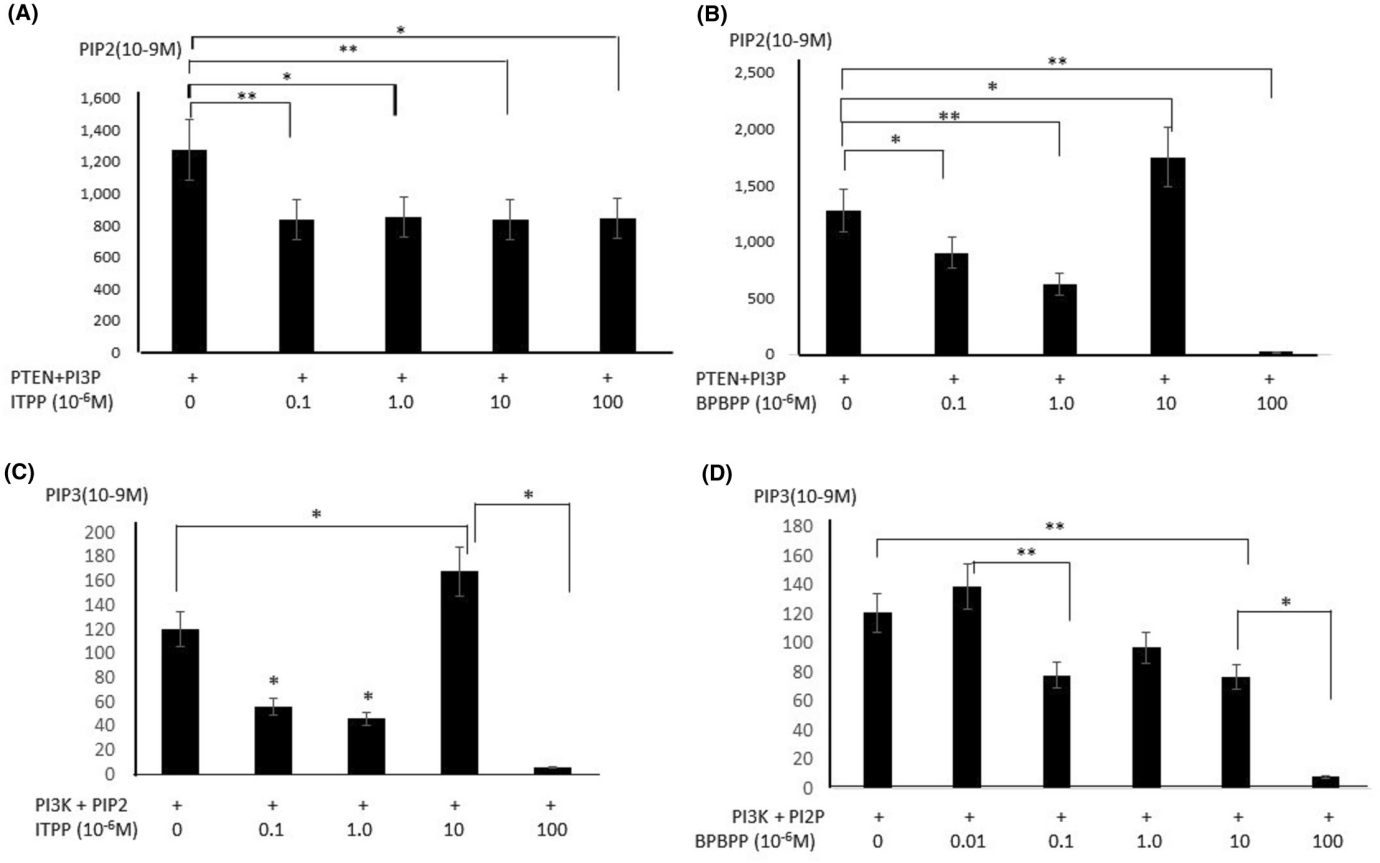
(A, B) Effect of ITPP and BPBPP on PTEN phosphatase activity on PIP3 by measurement of PIP2 production. (A) Soluble PIP3 (5.0 × 10^−6^ M) hydrolysis by PTEN (0.5 × 10^−6^ M), performed in the presence of variable concentrations (0.1 × 10^−6^ to 100.0 × 10^−6^ M) of ITPP measured by anti‐PIP2 antibodies binding competition. (B) Soluble PIP3 (5.0 × 10^−6^ M) hydrolysis by PTEN (0.5 × 10^−6^ M) performed in the presence of variable concentrations (0.1 × 10^−6^ to 100.0 × 10^−6^ M) of BPBPP measured by anti‐PIP2 antibodies binding competition. The data are the means of three separate experiments in triplicate. The data are the means ± SD of three separate experiments in triplicate (*p < 0.05, **p < 0.01). (C, D) Effect of ITPP and BPBPP on the activity of PI3‐Kinase on PIP2 by measuring PIP3 production. (C) Soluble PIP2 (5.0 × 10^−6^ M) phosphorylation by PI3K (0.5 × 10^−6^ M) was performed in the presence of variable concentrations (0.1 × 10^−6^ to 100.0 × 10^−6^ M) of ITPP measured by anti‐PIP3 antibodies binding competition. (D) Soluble PIP2 (5.0 × 10^−6^ M) phosphorylation by PI3K (0.5 × 10^−6^ M) performed in the presence of variable concentrations (0.1 × 10^−6^ to 100.0 × 10^−6^ M) of BPBPP measured by anti‐PIP3 antibodies binding competition. The data are the means of three separate experiments in triplicate.

The PTEN‐mediated hydrolysis of PIP3 into PIP2, measured by a competitive ELISA assay assessing the binding of anti‐PIP2 antibodies, is presented on Figure 3. This method was used to avoid the potential participation of phosphate residues from myoinositol derivatives in the above‐described green molybdate phosphate complex formation. During the PIP3 hydrolysis experiments by PTEN, in the conditions indicated by the manufacturer (5 × 10^−6^ M and 0.5 × 10^−6^ M), the tentative inhibitors: ITPP and BPBPP were tested in a concentration range from 0.1 × 10^−6^ M to 100 × 10^−6^ M. Figure 3A shows the inhibition of PTEN hydrolytic activity upon the presence of ITPP, while Figure 3B indicates a different mode of action revealed by BPBPP, which shows an activation effect at a 10 × 10^−6^ M concentration, which is in the range of the relative concentration used for the PIP3 substrate, and a strong inhibitory effect when a high BPBPP concentration (100 × 10^−6^M) was used. The data corroborates the previous direct measurement of PTEN phosphatase action described in Figure 2.

#### 3.2.3 Effect of myo‐inositol phosphate derivatives on PI3 kinase activity towards PIP2 and the production of PIP3

PI3‐Kinase activity on PIP2 for PIP3 production was measured by competitive ELISA assay assessing the binding of anti‐PIP3 antibodies as shown in Figure 3C, D. The PIP3 synthesis experiment was performed using PIP2, upon phosphorylation by PI3K, in the conditions indicated by the manufacturer (5 × 10^−6^ M and 0.5 × 10^−6^ M, respectively). The tentative inhibitors: ITPP and BPBPP were tested in a concentration range from 0.1 × 10^−6^ M to 100 × 10^−6^ M. Figure 3C shows the partial inhibition of PI3‐Kinase activity upon the presence of low ITPP concentration values (≤1.0 × 10^−6^ M), which is in the range of the relative concentration of the PIP2 substrate. The complexity of the mechanism is shown by the increased PI3K activity upon the addition of ITPP at a concentration of around 10 × 10^−6^ M, while ITPP inhibits the PI3K totally when its concentration reaches 100 × 10^−6^ M. Figure 3D reveals the different mode of action of BPBPP, which shows a partial inhibitory effect. A total inhibitory effect appears for a 100 × 10^−6^ M concentration of BPBPP.

### 3.3 Endothelial cell PTEN modulation and phospho‐Akt activation upon hypoxia/reoxygenation stress, the effects of ITPP and BPBPP

**FIGURE 4:**
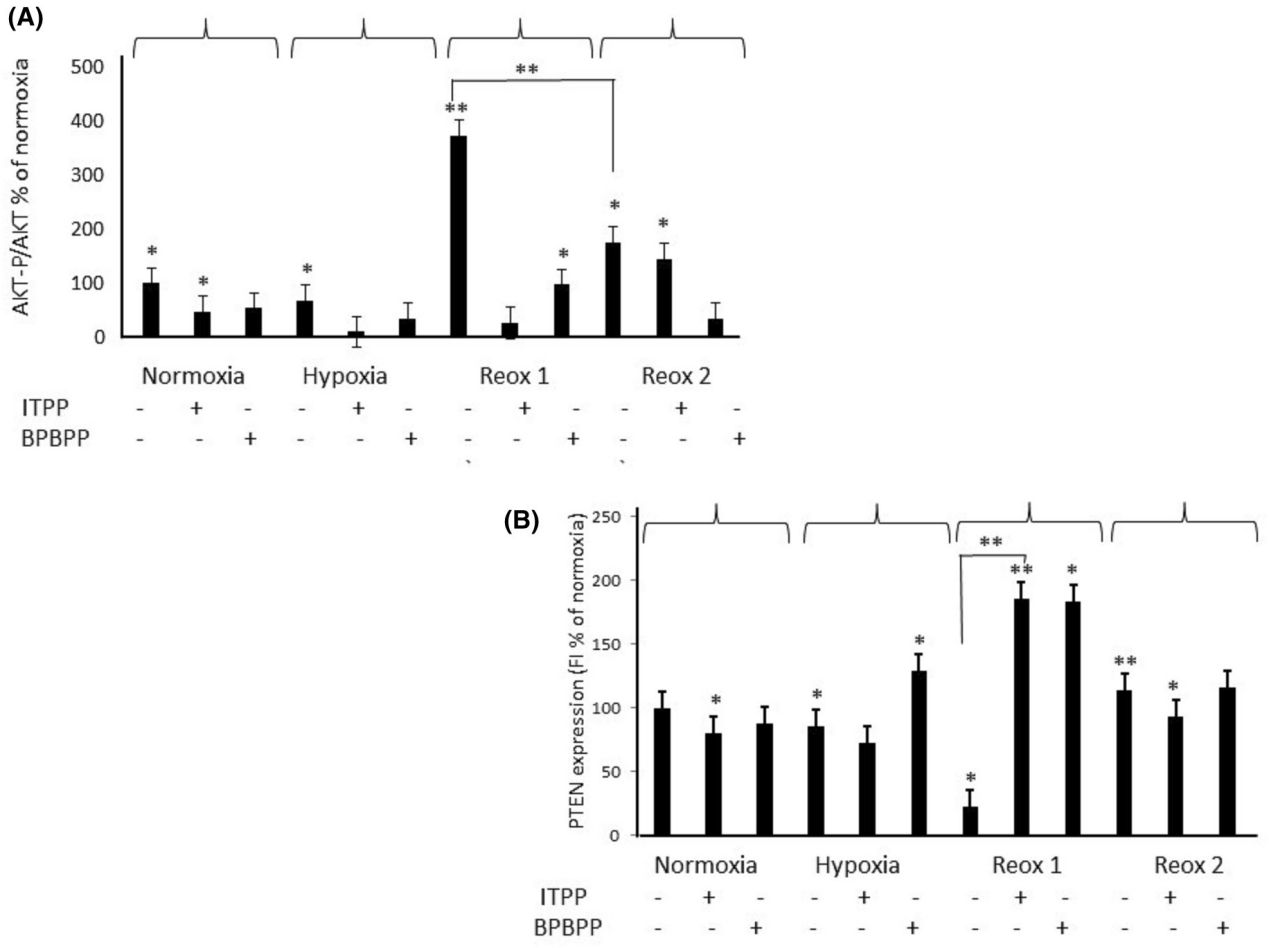
PTEN and p‐Akt modulation by hypoxia/reoxygenation stress in endothelial cells and the effects of ITPP and BPBPP protective treatment. Endothelial MLuMEC, FVB cells were cultured in parallel conditions in normoxia (19.5% O_2_) and hypoxia (1% O_2_) for 16 h. Reoxygenation was performed either directly (22 h) or after another 6 h in hypoxia in the presence of ITPP or BPBPP (20 × 10^−6^ M) for 16 h (Reox. 1), at which time ITPP and BPBPP were also applied to the cells in Reox. 2 conditions. (A) p‐Akt/Akt assessment by flow cytometry. The data represents the mean (+SD) from three experiments in triplicate (*p < 0.05, **p < 0.01). (B) PTEN assessment by flow cytometry. The data represents the mean (+SD) from three experiments in triplicate (*p < 0.05, **p < 0.01).

Since the effects of ITPP and its metabolite BPBPP appeared to operate on the activity of PTEN and PI3K differently, the elucidation of their mode of action required the investigation of their effect on the PTEN downstream signalling pathway. In order to assess whether ITPP and/or BPBPP act directly on the PTEN phosphatase regulatory pathway or primarily by PI3K inhibition, the regulation of the active form of Akt, that is phospho‐Akt, was estimated by the p‐Akt/Akt ratio as a function of the reaction to the pO_2_ variation, as shown in Figure 4A, B.

In normoxia, no clear effect of ITPP or BPBPP was observed, while in hypoxia, the endothelial cells from murine lungs MLuMEC, FVB^37^, ^38^, ^39^ showed no direct relation between the effects of ITPP or BPBPP on either p‐Akt/Akt (A) or PTEN (B). Contrary to this, reoxygenation for 22 h significantly reduced the expression of PTEN, which corresponded to a strong increase in the p‐Akt/Akt value.

When such reoxygenation occurred in the presence of both ITPP or BPBPP applied in prophylactic mode (Reox. 1), PTEN expression highly increased and was directly linked to p‐Akt/Akt reduction. Moreover, if the reoxygenation step occurred prior to the application of ITPP or BPBPP (Reox. 2) in curative mode, no direct relation between PTEN modulation and Akt activity was detected.

### 3.4 PIP3 binding characteristics to PTEN in vitro

**FIGURE 5:**
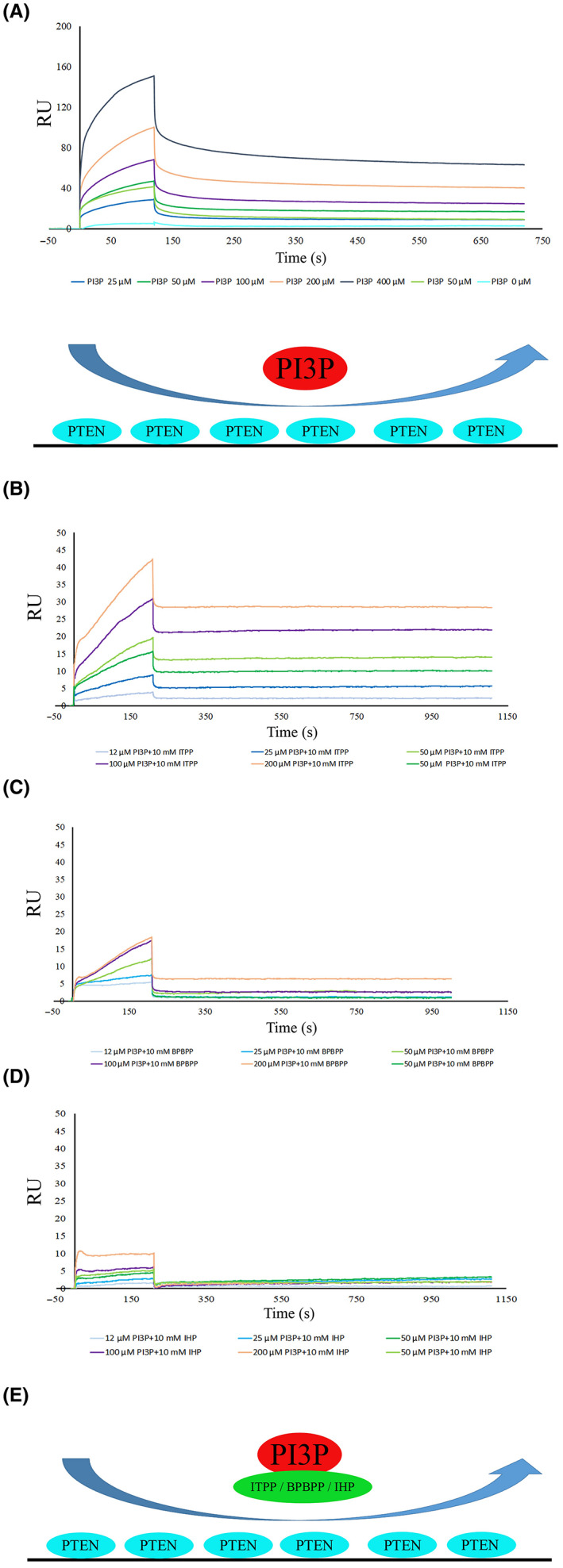
PIP3 binds PTEN with an equilibrium dissociation constant (*K*_D_), with values in the micromolar range and it is affected by ITPP, BPBPP and IHP. (A) PTEN was immobilized on a CM5 chip and its interaction with PIP3 (at concentrations ranging from 25–400 μM) and analysed using surface plasmon resonance (BIAcore T200). The details have been explained in the Materials and Methods section. (B–E) PTEN was immobilized on a CM5 chip and the interaction between PIP3 mixed with (B) ITPP, (C) BPBPP, and (D) IHP was analysed using surface plasmon resonance (BIAcore T200). The details have been provided in the Materials and Methods section.

To examine whether PIP3 binds to PTEN and how the analysed phosphates affect this interaction, PTEN was immobilized on a CM5 sensorchip and a series of concentrations of PIP3 was flown over the surface (Figure 5A). A kinetic analysis of the direct interactions between PTEN and PIP3 indicated an equilibrium dissociation constant (*K*_D_) of approximately 2.5 micromoles. The global association rate for this interaction was ~2.13 × 10^2^ 1/ms, whereas the dissociation was ~5.4 × 10^−4^ 1/s.

To evaluate the impact of ITPP/BPBPP/IHP and check its potential to modulate PIP3 and PTEN interaction, we analysed it using SPR, as described in the Materials and Methods section (Table 1). An injection of PIP3 as a mixture with ITPP or BPBPP or IHP (see Table 1, #3) revealed a significant change in PTEN‐PIP3 interactions (Figure 5B–E). First, in the case of the PIP3 and ITPP (20 mM) mixture, the association phase was clearly affected and seemed to be a two‐stage process. What is more, the formed complex was highly stable and almost no dissociation was observed (Figure 5B). This very slow dissociation rate of 5–8 × 10^−6^ 1/s, approximately 100 times slower than the pure PIP3‐PTEN complex, resulted in a calculated *K*_D_ in the nanomole range (20–30 nanomoles). Despite the visible similarities in the curvature of SPR sensorgrams for the interaction of PIP3 and PTEN in the presence of BPBPP, no clear kinetic data was obtained (Figure 5C). As for IHP, there was no PIP3‐PTEN interaction in the said mixture (Figure 5D).

To determine the stage on which ITPP or BPBPP or IHP impact the PIP3‐PTEN interaction, we pre‐injected 20 mM of ITPP or BPBPP over an immobilized PTEN, followed by an injection of PIP3 as the analyte (Table 1).

**FIGURE 6:**
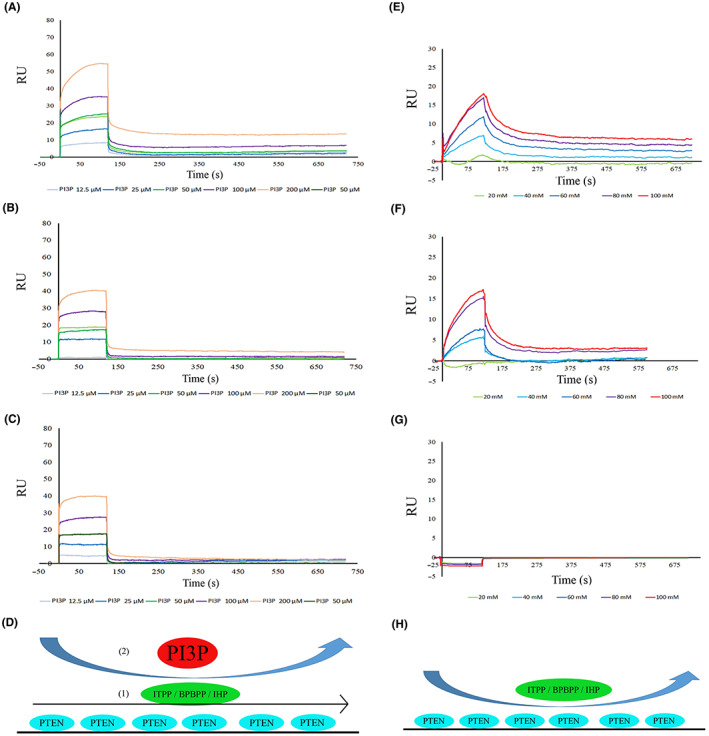
(A–D) PIP3 binding to PTEN is not affected by ITPP, BPBPP and IHP when they are preinjected. PTEN was immobilized on a CM5 chip and its interaction with (A) ITPP, (B) BPBPP and (C) IHP, followed by PIP3 and analysed using surface plasmon resonance (BIAcore T200). All the details have been explained in the Materials and Methods section. (E–H) ITPP, BPBPP and IHP interactions with PTEN analysed using SPR. PTEN was immobilized on a CM5 chip and its interaction with (E) ITPP, (F) BPBPP and (G) IHP and analysed by surface plasmon resonance using a BIAcore T200. All the details have been explained in the Materials and Methods section.

The pre‐injection of 20 mM ITPP or BPBPP and subsequent analysis of the PTEN–PIP3 interaction did not bring any important changes in the curves and *K*_D_. (Figure 6A–D).

It is worth mentioning that a direct interaction between PTEN and ITPP/BPBPP can be detected in our condition but only above 20 mM (Figure 6). Neither ITPP nor BPBPP were able to bind PTEN at a concentration below 20 mM; the interaction remained very weak with a *K*_D_ below instrument detection level even in higher concentrations (Figure 6E, F). IHP did not bind to PTEN at any investigated concentration (Figure 6G).

**FIGURE 7:**
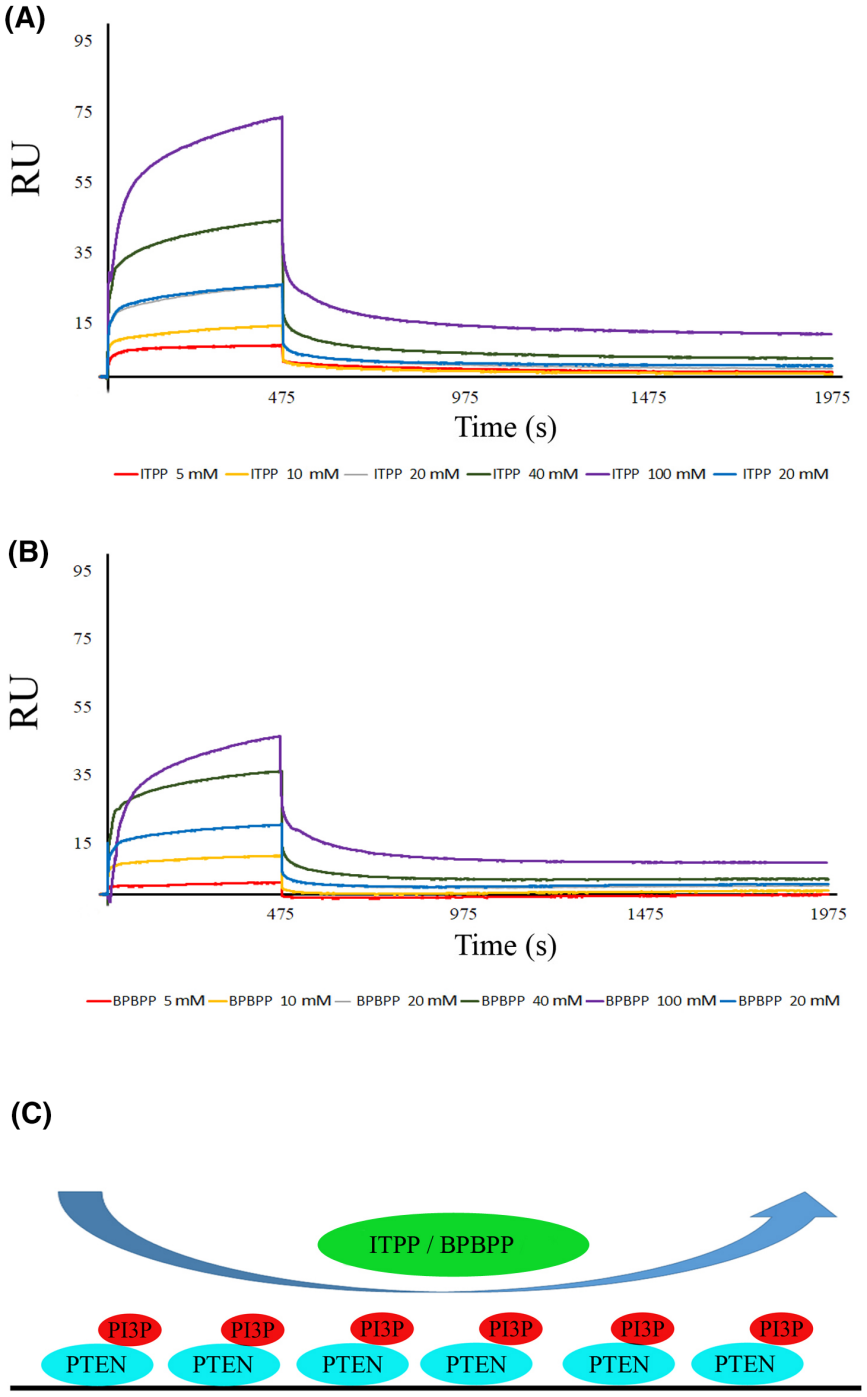
PTEN‐PIP3 complex strongly interacts with ITPP and BPBPP. PTEN was immobilized on a CM5 chip, complexed with 200 μM PIP3, and the interaction of such a complex with (A) ITPP and (B) BPBPP and analysed by surface plasmon resonance using a BIAcore T200. All the details have been explained in the Materials and Methods section.

Notably, when we investigated the binding of ITPP or BPBPP to complexed PIP3‐complexed PTEN, the binding got very strong as a nanomolar scale was observed (Figure 7), with a *K*_D_ of approximately 22 nanomoles for ITPP and 28 nanomoles for BPBPP.

## 4 DISCUSSION

The complex and diverse molecular mechanisms by which the PTEN tumour suppressor is able to act make it a highly valuable target for therapeutic strategies.^30^, ^33^, ^35^, ^36^, ^43^

Its actions are crucial as far as cell growth control is concerned, due to its upstream position in the growth activation pathway of PI3K/Akt/mTOR in the cytoplasm, as well as in tuning the p53 tumour suppressor in the nucleus. This goes together with its exocrine/paracrine regulatory effects on pathologic cells and the cells of the microenvironment, like the endothelial cells. Thus, PTEN activation presents a huge regulatory potential, which raises the need for a deeper knowledge to be gained of the PTEN reactions, mainly in view of its possible modes of activation.^31^, ^35^, ^36^

In the case of PTEN, being the main controller of angiogenesis, its active form in the endothelial cells is crucial for tumour angiogenesis normalization^32^ which, in turn, is vital to alleviate the reduced level of oxygen tension in the treatment of hypoxia‐dependent diseases, such as cancer, diabetes and cardiac and neurodegenerative diseases.^18^

As demonstrated by us, such activation is possible by myo‐inositol trispyrophosphate derivatives in the cancer,^19^ but also in cardiac disease^28^, ^44^ treatments. It also led us to hypothesize that PTEN activation is a complex process on which its further induced biological consequences are dependent. This prompted biochemical and biophysical approaches to be taken in order to understand the mode of PTEN interactions with ITPP and its metabolites as the BPBPP form, compared to inositol hexaphosphate (IHP). The biochemical approaches comparing ITPP and BPBPP reactions towards PTEN phosphatase activity confirmed their complex and concentration‐dependent effects. While ITPP was inhibitory for PTEN hydrolysis of PIP3 in the studied concentration range, BPBPP displayed an activation effect in a restricted concentration range; exactly inverse effects were obtained with respect to PI3‐Kinase activity towards PIP2.

Such behaviour appears to be of fundamental importance in view of future treatment applications, considering the therapeutic adjuvant effect of vessel normalization providing that the latter is stable, in order to avoid the pitfalls encountered by antiangiogenic strategies.

Therefore, a biophysical approach using the plasmon resonance technique was necessary to directly analyse the affinity and recognition properties of PTEN with the phosphoinositide derivatives and their effects on its natural hydrolytic reaction towards PIP3. This led to the evidence that ITPP and its metabolites are, individually, weak ligands for PTEN, but they work relatively efficiently and get to reach strong binding characteristics when they are present in association with PIP3. This allows them to be utilized in vivo, especially in the case of ITPP whose *K*_D_ upon interaction with PTEN was not measurable by SPR, but its range reached a nanomolar level after PTEN had reacted with PIP3. Indeed, this reaction explains the activation of PTEN upon in vivo treatment by ITPP since it reproduces the in situ conditions. In the cell, PTEN is a phosphatase that maintains the PIP3 to PIP2 balance for activation signal control, thus, it is always in the presence of PIP3. Consequently, in natural in situ conditions, PTEN, in the presence of PIP3, is capable of binding ITPP with a high affinity and this, in turn, promotes an increased affinity of PTEN for PIP3, as demonstrated herein.

Thus, the activity of PTEN in the presence of ITPP favours the production of PIP2, thus, reducing the action of PI3K. Moreover, our biochemical data show that ITPP is an inhibitor of PI3K, making its application in cancer cell growth control very promising. Moreover, these data also confirm the potential of in vivo ITPP injections for angiogenesis normalization, as PTEN is a vital molecule whose activity may ensure proper and organized angiogenesis.

## AUTHOR CONTRIBUTIONS

**Krzysztof Grzymajlo:** Investigation (equal); methodology (equal); writing – original draft (supporting); writing – review and editing (equal). **Bouchra El Hafny‐Rahbi:** Data curation (equal); investigation (equal); methodology (equal); writing – original draft (supporting). **Claudine Kieda:** Conceptualization (equal); formal analysis (equal); funding acquisition (equal); methodology (equal); supervision (lead); writing – original draft (lead); writing – review and editing (equal).

## FUNDING INFORMATION

The research was partly funded by Military Institute of Medicine intramural grant no. 1/9005 (520) and National Science Centre Poland grant no. OPUS 2016/23/B/NZ6/02542.

## CONFLICT OF INTEREST STATEMENT

The authors confirm that there are no conflicts of interest.

## Data Availability

Data available on request from the authors.
